# The Illness Experience of Veterans With Dementia: Perceived Measures Related to Depressive Symptoms

**DOI:** 10.1093/geroni/igab046.3126

**Published:** 2021-12-17

**Authors:** Amanda MacNeil, Katherine Judge, Kate McCarthy, David Bass

**Affiliations:** 1 Cleveland State University, Fairview Park, Ohio, United States; 2 Cleveland State University, Cleveland, Ohio, United States; 3 Benjamin Rose Institute on Aging, Benjamin Rose Institute on Aging, Ohio, United States; 4 Benjamin Rose Institute on Aging, Cleveland, Ohio, United States

## Abstract

Recent work has examined how individuals with dementia (IWDs) experience their illness, although few studies have looked at IWDs who report heightened depressive symptoms, a key well-being outcome. Stressing the ability of IWDs to self-report and guided by the Stress Process Model for Individuals with Dementia, this study examined the relationships between depressive symptoms and various aspects of the illness experience including objective cognition, perceived memory difficulty, perceived functional difficulty, and dyadic relationship strain. The sample includes IWDs with mild to severe dementia who are veterans (N=69). Significant positive correlations emerged between depressive symptoms and several measures of the illness experience: perceived cognition (r=.48, p<.001), perceived function (r =.43, p<.001), and dyadic relationship strain (r=.32, p=.01). In contrast, objective cognition, measured by a modified version of the Blessed Orientation Memory Concentration test, was not significant (r =-.06, p=.63). A multiple regression found the total variance explained by all independent variables was 32% (R2=.32, F(4,68)=7.58, p<.001), with perceived memory difficulty (B=.26, p<.01) and dyadic relationship strain (B=.25, p=.04) accounting for unique and significant variance in depressive symptoms. A mediation analysis indicated perceived memory difficulty fully mediated the relationship between perceived functional difficulty and depressive symptoms. Findings highlight the importance of IWDs perceptions of their illness experience for psychosocial well-being outcomes, such as depressive symptoms. Findings add to the literature by showing the importance of IWDs perceptions of their illness and their impact on well-being outcomes. Results also demonstrate the utility and feasibility of including self-reported data from IWDs in research studies.

